# Modeling methicillin-resistant *Staphylococcus aureus* (MRSA) acquisitions in the intensive care unit with different staffing levels and finite direct-care tasks

**DOI:** 10.1017/ash.2023.362

**Published:** 2023-09-29

**Authors:** Stephanie Johnson, Matthew Mietchen, Eric Lofgren

## Abstract

**Background:** Modeling is a cost-effective way to evaluate interventions pertaining to hospital infection acquisitions, such as staffing levels. Increasing the number of nurses in an intensive care unit affects rates of HAI transmission. The way compartmental models are often formulated assumes that there is a never-ending series of tasks for workers to perform. Our previous models used a baseline of 1:3 nurse:patient ratio, and we kept the number of tasks the same across staffing ratios. We wanted to understand how having a finite number of tasks, using this baseline number, across staffing levels affected HAI acquisitions. **Methods:** We used a stochastic mathematical model of methicillin-resistant *Staphylococcus aureus* (MRSA) to study the impact of changes in staffing and a finite pool of tasks on hospital-associated acquisitions. For a 15-bed intensive care unit (ICU), we have 1 intensivist, and we set the nurse:patient ratios at 1:1, 1:1.5, 1:2.5, 1:3, 1:5, and 1:7.5, to represent the extreme ends of staffing levels and more moderate values in line with critical care society guidelines. Each model was run 1,000 times. The outcome of each scenario is the median number of hospital-associated MRSA acquisitions in 1 year from those 1,000 runs. **Results:** Treating the 1:3 nurse:patient ratio as the baseline, with 45 MRSA acquisitions per year, increasing the number of nurses from 5 to 6 (moving to a 1:2.5 nurse:patient ratio) had a relative risk (RR) of 0.77, suggesting that a small change in nurse staffing levels might have an outsized impact on rates. More dramatic changes had correspondingly larger swings in MRSA acquisition rates, with 1:1 nurse:patient ratio scenarios having an RR of 0.17, and at the other extreme, a 1:7.5 nurse:patient ratio having an RR of 4.66. Comparing the infinite to finite models, the ratios with more nurses had lower acquisition rates, with decreases ranging from 20% to 50%. Ratios with fewer nurses in the ICU showed 100%–400% increases in the number of acquisitions. All results were statistically significant. **Conclusions:** As nurse:patient ratios go up, the burden of direct-care tasks fall on fewer people, which has a direct impact on HAI rates. Our model demonstrates this hypothesis. Therefore, appropriate staffing should be considered in infection control guidelines, and the cost of staffing should be weighed against its impact on infection prevention as well as other areas of patient care. In this study, we considered only the impact from changes in contact patterns emerging from different staffing levels. Further insights may exist when considering other outcomes that also accompany increased staffing.

**Disclosures:** None

